# EHMTI-0039. Different efficacy of acute migraine therapies for migraine with aura versus without aura

**DOI:** 10.1186/1129-2377-15-S1-G26

**Published:** 2014-09-18

**Authors:** J Møller Hansen, PJ Goadsby, A Charles

**Affiliations:** 1 Department of Neurology: Headache Research and Treatment Program, University of California Los Angeles (UCLA), Los Angeles, USA; 2 Department of Neurology: Headache Group, University of California San Francisco (UCSF), San Francisco, USA

## Objective

To determine if acute migraine treatment outcome is different in patients with migraine with aura compared those with migraine without aura.

## Methods

We extracted treatment outcome for migraine with and without aura from large-scale randomized clinical trials of two different drugs with proven efficacy in migraine treatment; sumatriptan and inhaled dihydroergotamine (DHE).

## Results

The pain free rates 2 h post-dose for sumatriptan 100 mg were significantly higher in patients treating attacks without aura (32%), compared to the group who treated attacks with aura (24%),(P < 0.001). For DHE the 2 h pain free rates did not differ between patients treating attacks without aura (29.4%) compared to those who treated attacks with aura (27.2%; P = 0.65), see figure. The NNT to achieve 2 h pain free for sumatriptan was 4.3 for attacks without aura and 6.2 for attacks with aura. For DHE, NNT for 2 h pain free was 5.8 for attacks without aura and 5.0 for attacks with aura.

**Figure 1:**
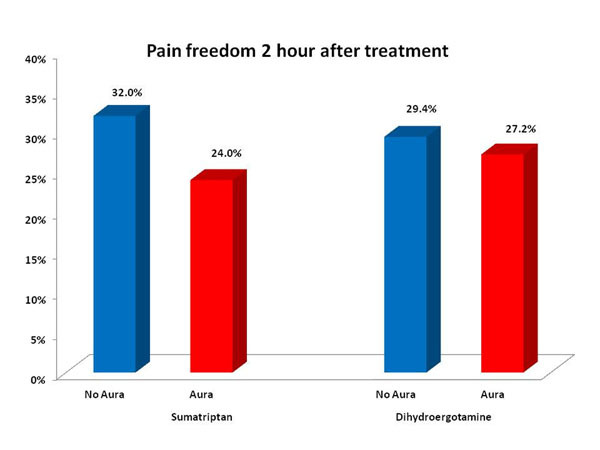


## Conclusion

These data indicate that sumatriptan is less effective as acute therapy for migraine attacks with aura compared to attacks without aura. Inhaled DHE, by contrast, had similar efficacy for migraine attacks with and without aura. Different responses of migraine with vs. without aura to acute therapies may provide insight into underlying mechanisms of the disorder. In addition, these different responses may have implications regarding design of clinical trials, and may influence the choice of acute therapies for different types of migraine attacks.

No conflict of interest.

